# Relationship of Hydroxychloroquine and Ophthalmic Complications in Patients with Type 2 Diabetes in Taiwan

**DOI:** 10.3390/ijerph18158154

**Published:** 2021-08-01

**Authors:** Hung-Chih Chen, Hung-Yu Lin, Michael Chia-Yen Chou, Yu-Hsun Wang, Pui-Ying Leong, James Cheng-Chung Wei

**Affiliations:** 1 Department of Ophthalmology, Show Chwan Memorial Hospital, Changhua 500, Taiwan; b101098004@tmu.edu.tw (H.-C.C.); anthonyhungyulin@hotmail.com (H.-Y.L.); mike_chou328@hotmail.com (M.C.-Y.C.); 2 Department of Optometry, Chung Shan Medical University, Taichung 402, Taiwan; 3 Institute of Medicine, Chung Shan Medical University, Taichung 402, Taiwan; 4 Department of Medical Research, Chung Shan Medical University Hospital, Taichung 402, Taiwan; cshe731@csh.org.tw; 5 Department of Medicine, Chung Shan Medical University Hospital, Taichung 402, Taiwan; 6 Division of Allergy, Immunology and Rheumatology, Department of Internal Medicine, Chung Shan Medical University Hospital, Taichung 402, Taiwan; 7 PhD Program in Business, Feng Chia University, Taichung 407, Taiwan; 8 Graduate Institute of Integrated Medicine, China Medical University, Taichung 402, Taiwan

**Keywords:** hydroxychloroquine, diabetic retinopathy, retinal pigment epithelium

## Abstract

The purpose of this study is to evaluate the relationship between hydroxychloroquine (HCQ) and diabetic retinopathy (DR) via the national health insurance research database (NHIRD) of Taiwan. All patients with newly diagnosed type 2 diabetes (*n* = 47,353) in the NHIRD (2000–2012) were enrolled in the study. The case group consists of participants with diabetic ophthalmic complications; 1:1 matching by age (±1 year old), sex, and diagnosis year of diabetes was used to provide an index date for the control group that corresponded to the case group (*n* = 5550). Chi-square test for categorical variables and Student’s t-test for continuous variables were used. Conditional logistic regression was performed to estimate the adjusted odds ratio (aOR) of DR. The total number of HCQ user was 99 patients (1.8%) in the case group and 93 patients (1.7%) in the control group. Patients with hypertension (aOR = 1.21, 95% CI = 1.11–1.31) and hyperlipidemia (aOR = 1.65, 95% CI = 1.52–1.79) significantly increased the risk of diabetic ophthalmic complications (*p* < 0.001). Conversely, the use of HCQ and the presence of rheumatoid diseases did not show any significance in increased risk of DR. HCQ prescription can improve systemic glycemic profile, but it does not decrease the risk of diabetic ophthalmic complications.

## 1. Introduction

Hydroxychloroquine (HCQ) is a unique medication commonly prescribed in rheumatology and dermatology [1]. In recent decades, several literatures have proven that HCQ is effective in minimizing the risk of new onset diabetes and improving metabolic profiles such as fasting blood sugar, insulin sensitivity, hemoglobin A1c (HbA1c) and lipid profiles [2,3,4,5,6,7,8,9]. These benefits were reported in patients with autoimmune diseases such as systemic lupus erythematosus [10], ankylosing spondylitis, rheumatoid arthritis, psoriasis [11,12] and Sjogren’s syndrome [8].

Diabetes mellitus is a rapidly growing public health concern across the world. The pathophysiology is related to chronic hyperglycemia with impaired metabolism due to insulin secretion dysfunction and/or peripheral tissue insulin resistance [13]. According to the World Health Organization (WHO) and the International Diabetes Federation (IDF), the prevalence of DM increased from 108 million (4.7%) in 1980 to 463 million (9.3%) in 2019, and it is estimated to be 700 million (10.9%) by 2045 [14,15]. Diabetes causes substantial morbidity and mortality, increasing the risk of cardiovascular disease, blindness, kidney failure and lower limb amputation [16]. Diabetic retinopathy is a disease caused by microvascular complications resulting from chronic diabetes and remains the leading cause of blindness worldwide. Associated risk factors inducing DR include hyperglycemia, hypertension, dyslipidemia, ethnics, duration of diabetes, pregnancy, puberty and previous cataract surgery [17]. Recently, it is thought to be influenced by disruption of the blood-retinal barriers (BRBs), which is composed of retinal endothelial cells and retinal pigment epithelium (RPE) [18,19,20].

Retinal pigment epithelium constitutes a monolayer of cuboidal, polarized cells situated between the photoreceptor cells and choroid [21,22]. RPE plays an essential functional role in the maintenance of retinal homeostasis and the health of photoreceptors [23]. Impairment of the RPE causes many inherited and acquired diseases, which may result in permanent blindness [24]. Studies have shown that HCQ disrupts RPE metabolism, resulting in retinal toxicity, especially in the macula of patients under long-term use of HCQ [25]. Patients with more serious side effects may experience color vision changes or paracentral scotomas with corresponding external limiting membrane loss, disruption of the ellipsoid zone, parafoveal thinning of the outer nuclear layer, and RPE damage [26,27].

Considering the conflicting effects associated with HCQ, we aimed to evaluate the relationship between HCQ and diabetic retinopathy by analyzing the national health insurance research database (NHIRD) of Taiwan. Other systemic comorbidities were also examined in the multivariate analysis model.

## 2. Materials and Methods

### 2.1. Data Source

The study is a nested case-control study incorporating data from the NHIRD, which enrolled almost 99% of a population of 23 million beneficiaries in Taiwan. The database comprises all insurance claims, including outpatient visits, emergency, and hospitalization. One million subjects were sampled from the 23 million beneficiaries dating from 1999 to 2013. The sampled database contains the de-identified medical records required for medical research and the study was approved by the Institutional Review Board of Chung Shan Medical University Hospital (Registration Number: CSMUH CS15134). The diagnostic codes were recorded according to the International Classification of Diseases, Ninth Revision, Clinical Modification (ICD-9-CM).

### 2.2. Patient Selection

The inclusion criteria of the study population consisted of newly diagnosed type 2 diabetes (ICD-9-CM = 250) and use of hypoglycemic agents within 30 days from 2000 to 2012; the exclusion criteria consisted of type 1 diabetes (ICD-9-CM = 250.×1, 250.×3) and newly diagnosed ophthalmic complications after the diagnosis of diabetes within one year. The case group were patients diagnosed with ophthalmic complications, including diabetes with ophthalmic manifestations (ICD-9-CM = 250.5), and diabetic retinopathy (ICD-9-CM = 362.0) with at least two outpatient visits or one hospitalization after diagnosis of diabetes at least one year apart. The control group were diabetic patients without previous diagnosis of ophthalmic complications. The index date was defined as the date of the patient’s first record of ophthalmic complications. Moreover, 1:1 matching by age (±1 year old), sex, and duration of diabetes was performed to provide an index date for the control group that corresponded to the case group.

### 2.3. Hydroxychloroquine and Covariates

The cumulative days of hydroxychloroquine was calculated from the first date of diabetes to the index date. The baseline characteristics were age, gender, hypertension (ICD-9-CM = 401–405), hyperlipidemia (ICD-9–CM = 272.0–272.4), rheumatoid arthritis (ICD-9-CM = 714.0), ankylosing spondylitis (ICD-9-CM = 720.0), systemic lupus erythematosus (ICD-9-CM = 710.0), and Sjogren’s syndrome (ICD-9-CM = 710.2). The comorbidities included were required to be diagnosed within one year before the index date, with at least two outpatient visits or one hospitalization. Since most patients in the NHIRD were Taiwanese, race was not considered as a covariate.

### 2.4. Statistical Analysis

To compare the characteristics of the case and control groups, Chi-square test for categorical variables and Student’s t- test for continuous variables were used. Conditional logistic regression was used to estimate the odds ratio of effect of hydroxychloroquine. Moreover, the dose effect of hydroxychloroquine was estimated by counting the cumulative days of use. A sensitivity analysis matching hypertension and hyperlipidemia was conducted. Statistical analysis was performed with the use of SPSS version 18.0 (SPSS Inc., Chicago, IL, USA).

## 3. Results

**Figure 1:**
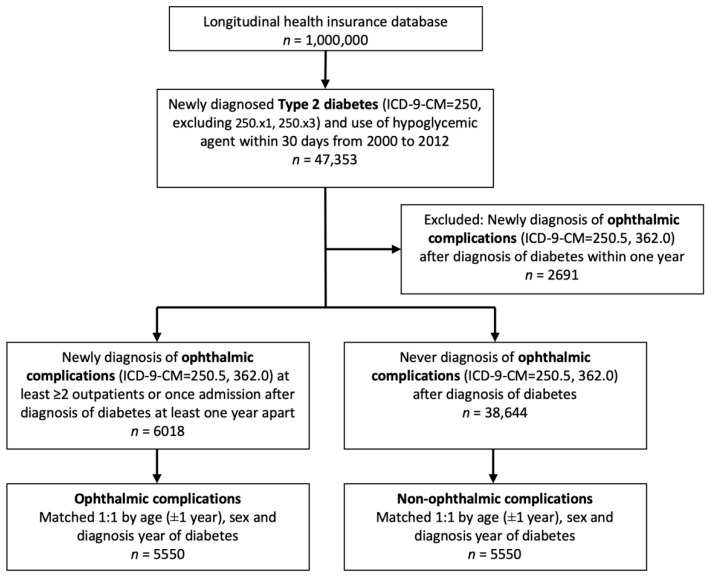
Flowchart of type 2 diabetes patient enrollment with and without ophthalmic complications.

The flowchart of patient selection is shown in Figure 1. After inclusion and matching, 5550 patients were selected in each group. There was no difference between the age and gender in the case and the control groups after matching. The demographics of diabetic patients are listed in Table 1, separated by the presence of ophthalmic complications.

The total number of HCQ user was 99 patients (1.8%) in the case group and 93 patients (1.7%) in the control group. The median age was 60.0 ± 11.3 and 60.1 ± 11.3 years and the sex ratio between female and male was 48.6% and 51.4% in two groups, respectively. The ratio of underlying medical diseases in the two groups were 65.8%/60.8% (*p* < 0.001) in hypertension; 57.9%/46.0% (*p* < 0.001) in hyperlipidemia; 1.4%/1.6% (*p* = 0.345) in rheumatoid arthritis; 0.4%/0.3% (*p* = 0.342) in ankylosing spondylitis; 0.1%/0.1% (*p* = 0.365) in systemic lupus erythematosus; and 0.9%/0.6% (*p* = 0.121) in Sjogren’s syndrome.

The conditional logistic regression of risk of developing ophthalmic complications compared with the reference group (without HCQ prescription) revealed significantly higher risk in the group with hypertension (adjust odds ratio (aOR) = 1.21, 95% confidence interval (C.I.) = 1.11–1.31, *p* < 0.001) and hyperlipidemia (aOR = 1.65, 95% C.I. = 1.52–1.79, *p* < 0.001). There was no significance in groups with HCQ prescription, rheumatoid arthritis, ankylosing spondylitis, systemic lupus erythematosus and Sjogren’s syndrome (Table 2). The sensitivity analysis was performed as supplement Figure S1, Tables S1 and S2 and there was no significance in groups with HCQ prescription and ophthalmic complications.

Subgroup analysis of risk of ophthalmic complications revealed there was no significance among all subgroups, regardless of the use of HCQ (Table 3).

Risk of ophthalmic complications related to the cumulative days of HCQ revealed no significance in all groups regardless of the duration of HCQ prescription (Table 4).

## 4. Discussion

In our study, we did not find a significant correlation between HCQ and the development of diabetic ophthalmic complications. Our finding was in conflict with the evidence displayed in previous literatures, which showed a positive relationship between HCQ and systemic glycemic control [2,3,4,5,6,7,8,9].

HCQ is considered toxic to the retina due to accumulation in the RPE, causing serious symptoms in long-term HCQ users. However, the mechanism of hydroxychloroquine retinal toxicity has yet to be totally understood [28]. Studies have revealed that HCQ affects the metabolism of retinal cells by binding to melanin in the RPE [29]. At the same time, the function of photoreceptor and RPE plays a key role in the progression of diabetic retinopathy [18].

However, HCQ has also shown to decrease the risk of diabetes and improve metabolic profile and hyperglycemia associated inflammation [2,3,4,5,6,7,8,9]. Insulin sensitivity and insulin resistance are improved because HCQ can inhibit the degradation of insulin [30], reduce hyperglycemia associated inflammation [31] and activate protein kinase β (Akt) resulting in increased glucose uptake and glycogen synthesis [32]. Furthermore, HCQ stabilizes intracellular lysosomes by decreasing the decomposition of the internalized insulin receptor complex [33]. HCQ is an acidotrophic agent, thus intracellular pH is raised with increasing intracellular concentration of HCQ. This causes inactivation of the proteolytic enzyme insulinase, which is responsible for the degradation of insulin, resulting in the recirculation of a massive proportion of insulin in the active form [34]. Moreover, HCQ can reduce the risk of cardiovascular morbidity [35,36,37], with the hypothesis of improvement in atherosclerosis [38] and anti-platelet properties through the arachidonic acid pathway [39]. The possible antidiabetic and arterial protective mechanisms of HCQ are gradually proposed, but further mechanistic, efficacy, and safety-related preclinical and clinical studies are warranted to verify the therapeutic role of this medication [9].

Due to the conflicting effects of HCQ on the retina, we designed this study to determine if HCQ has a preventive or detrimental effect on the development of diabetic retinopathy. In our study, we revealed that patients with hypertension (aOR = 1.21, 95% C.I. = 1.11–1.31) and hyperlipidemia (aOR = 1.65, 95% C.I. = 1.52–1.79) were strongly associated with the risk of diabetic ophthalmic complications (Table 1). The result was compatible with the previous studies indicating these two known comorbidities [17]. The same result was also shown in Table 2. However, HCQ prescription had no significant effect in decreasing the risk of diabetic ophthalmic complications. This result was inconsistent with the evidence proposed in previous literatures, which showed that HCQ had antidiabetic properties [2,3,4,5,6,7,8,9]. Rosa et al. reported that diabetes increases inflammation and oxidative stress and thus influences cellular metabolism, including autophagy, establishing a degree of cellular apoptosis and the progression of DR [40]. Chen et al. surveyed that HCQ inhibit autophagy of RPE and further aggravated the senescence of it, accompanied by an increase in oxidant species [41]. As a result, HCQ has a negative effect in cellular metabolism which may further exacerbate DR.

In the subgroup analysis of risk of ophthalmic complications (Table 3), we also revealed that HCQ had no significant effect on decreasing the risk of diabetic retinopathy in any subgroups. Interestingly, there is an increased odds ratio of diabetic complications in patients with hyperlipidemia under HCQ prescription (OR = 1.33, 95% C.I. = 0.88–2.01, *p* = 0.170). The result suggests that HCQ may induce progression of DR, especially in patients with an abnormal lipid profile. Chen et al. reported that HCQ aggravates RPE dysfunction due to oxidative stress mediated by lipid metabolism, contributing to high glucose-induced senescence [41]. This finding suggests an increased risk of progression of DR in diabetic patients with dyslipidemia treated with HCQ. In regard to the cumulative days of effect that HCQ has on diabetic retinopathy (Table 4), the duration of HCQ use, whether short (<90 days), long (>90 days), or as a continuous variable, has no significant effect on the development of diabetes related ophthalmic complications.

Although HCQ has proven to have systemic antidiabetic properties, our study did not show that it has the same effect in preventing diabetic retinopathies. We postulate that HCQ may influence the metabolism and pathophysiology of the retina, especially RPE, which may encourage the progression of DR. Diabetic retinopathy is a multi-factorial disease, controlled not only by blood sugar or hemoglobin A1c. Through this study, we wanted to evaluate the role of HCQ in diabetic retinopathy, which is clinically relevant for many rheumatoid patients with diabetes under prolonged use of HCQ.

There are some limitations in this study in regard to the national health insurance research database. First, we could not confirm the specificity and consistency of diabetic ophthalmic complications since it was diagnosed and coded by different ophthalmologists. Additionally, the coding rate may be underestimated due to low ophthalmologic screening rate of HCQ users in Taiwan [42]. Secondly, we could not evaluate the severity of DR with the current data. More detailed stages of DR should also be considered for evaluating the effect of HCQ on the severity of ophthalmic complication. Besides, different dose and type of antidiabetic medication combined with HCQ may have provided varying degrees of treatment on DR. Hsia et al. reported that HCQ improved glucose regulation when used as a third-line agent added to metformin and a sulfonylurea [43]. More randomized controlled trial is warranted to establish the efficacy of combining drugs with HCQ on treating diabetic retinopathy in the future. Finally, detailed information of HCQ prescription was not available in the database. The American Academy of Ophthalmology (AAO) guidelines (2011 and 2016) claimed that it is a major risk factor that increases the risk of retinal toxicity after 5 years of HCQ therapy [44,45]. In our study, the number of patients under HCQ therapy longer than 5 years was too small to analyze. Besides, the 2011 AAO guidelines stated that a cumulative dose of >1000 g increases the risk of retinopathy (equating to 6.85 years of treatment at 400 mg, and 13.7 years of treatment at 200 mg) [44]. The exact cumulative dose of HCQ was not obtained in this study.

HCQ is a historical medication used broadly and commonly even in modern medicine. The research of HCQ retinopathy and systemic antidiabetic property of HCQ are gradually being established. This is a preliminary and novel study to explore the relationship between HCQ and diabetic ophthalmic complications. Our result provides some clinical insights for ophthalmologists and physicians on the management of DR in patients with concomitant diseases requiring HCQ prescription.

## 5. Conclusions

HCQ is commonly prescribed for rheumatic and dermatologic diseases in Taiwan, and many of these patients have diabetes. Although HCQ can improve glycemic profiles systemically with careful use, it does not seem to decrease the risk of diabetic ophthalmic complications. The protective and injurious mechanism of HCQ on diabetic retinopathy needs to be further investigated.

## Figures and Tables

**Table 1:** Demographic characteristics of diabetic patients.

Variables	Case		Control		
	*n*	%	*n*	%	*p*-Value
Total	5550		5550		
Subgroups					
HCQ	99	1.8	93	1.7	0.662
Age					0.743
<40	202	3.6	195	3.5	
40–65	3520	63.4	3491	62.9	
≥65	1828	32.9	1864	33.6	
Mean ± SD	60.0 ± 11.3		60.1 ± 11.3		0.643
Sex					1
Female	2695	48.6	2695	48.6	
Male	2855	51.4	2855	51.4	
Hypertension	3651	65.8	3372	60.8	<0.001
Hyperlipidemia	3213	57.9	2553	46.0	<0.001
Rheumatoid arthritis	76	1.4	88	1.6	0.345
Ankylosing spondylitis	23	0.4	17	0.3	0.342
Systemic lupus erythematosus	4	0.1	7	0.1	0.365
Sjogren’s syndrome	48	0.9	34	0.6	0.121

*n*: sample size; HCQ: hydroxychloroquine; SD: standard deviation.

**Table 2:** Conditional logistic regression of risk of ophthalmic complications.

Variables	cOR	95% C.I.	*p*-Value	aOR ^†^	95% C.I.	*p*-Value
HCQ						
No	Reference			Reference		
Yes	1.07	0.80–1.42	0.663	1.10	0.81–1.49	0.526
Hypertension	1.27	1.17–1.38	<0.001	1.21	1.11–1.31	<0.001
Hyperlipidemia	1.68	1.55–1.82	<0.001	1.65	1.52–1.79	<0.001
Rheumatoid arthritis	0.86	0.64–1.17	0.349	0.79	0.57–1.10	0.163
Ankylosing Spondylitis	1.35	0.72–2.53	0.345	1.28	0.68–2.42	0.448
Systemic Lupus Erythematosus	0.57	0.17–1.95	0.372	0.47	0.13–1.66	0.239
Sjogren’s syndrome	1.42	0.91–2.22	0.119	1.44	0.92–2.27	0.114

cOR: crude odds ratio; C.I.: confidence interval; aOR: adjusted odds ratio; HCQ: hydroxychloroquine. ^†^ Adjusted for HCQ, hypertension, hyperlipidemia, rheumatoid arthritis, ankylosing spondylitis, systemic lupus erythematosus, and Sjogren’s syndrome.

**Table 3:** Subgroup analysis of risk of ophthalmic complications.

Variables	HCQ		Non-HCQ				
	*n*	Complication	*n*	Complication	OR	95% C.I.	*p*-Value
Age ^a^							
<40	6	5	391	197	6.63	0.76–58.17	0.088
40–65	116	59	6895	3461	1.01	0.70–1.47	0.944
≥65	70	35	3622	1793	1.00	0.62–1.62	0.993
Sex ^b^							
Female	151	72	5239	2623	1.01	0.71–1.43	0.971
Male	41	27	5669	2828	1.75	0.90–3.39	0.096
Hypertension ^c^							
No	70	31	4007	1868	0.99	0.59–1.66	0.974
Yes	122	68	6901	3583	1.18	0.81–1.71	0.397
Hyperlipidemia ^d^							
No	87	35	5247	2302	0.85	0.54–1.36	0.507
Yes	105	64	5661	3149	1.33	0.88–2.01	0.170
Rheumatoid arthritis ^e^							
No	149	81	10,787	5393	1.14	0.82–1.58	0.440
Yes	43	18	121	58	0.88	0.42–1.86	0.736
Ankylosing spondylitis ^f^							
No	189	97	10,871	5430	1.10	0.81–1.49	0.551
Yes	3	2	37	21	0.92	0.07–12.33	0.949
Systemic lupus erythematosus ^g^							
No	185	96	10,904	5450	1.09	0.81–1.48	0.571
Yes	7	3	4	1	NA	NA	NA
Sjogren’s syndrome ^h^							
No	175	92	10,843	5410	1.17	0.86–1.59	0.329
Yes	17	7	65	41	0.43	0.11–1.64	0.219

*n*: patient number of subgroups; Complication: patient number with ophthalmic complication; HCQ: hydroxychloroquine; OR: odds ratio; C.I.: confidence interval; NA: not applicable. ^a^: adjusted for hypertension, and hyperlipidemia. ^b^: adjusted for hypertension, hyperlipidemia, rheumatoid arthritis, ankylosing spondylitis, systemic lupus erythematosus, and Sjogren’s syndrome. ^c^: adjusted for hyperlipidemia, rheumatoid arthritis, ankylosing spondylitis, systemic lupus erythematosus, and Sjogren’s syndrome. ^d^: adjusted for hypertension, rheumatoid arthritis, ankylosing spondylitis, systemic lupus erythematosus, and Sjogren’s syndrome. ^e^: adjusted for hypertension, hyperlipidemia, ankylosing spondylitis, and Sjogren’s syndrome. ^f^: adjusted for hypertension, hyperlipidemia, rheumatoid arthritis, systemic lupus erythematosus, and Sjogren’s syndrome. ^g^: adjusted for hypertension, hyperlipidemia, rheumatoid arthritis, ankylosing spondylitis, and Sjogren’s syndrome. ^h^: adjusted for hypertension, hyperlipidemia, rheumatoid arthritis, ankylosing spondylitis, and systemic lupus erythematosus.

**Table 4:** Risk of ophthalmic complications with different cumulative days of HCQ.

Cumulative Days of HCQ	*n*	Complicaion	aOR ^†^	95% C.I.	*p*-Value
0 *	10,908	5451	Reference		
<90	105	53	1.06	0.71–1.57	0.783
≥90	87	46	1.17	0.74–1.83	0.501
HCQ (continuous variable)					0.499

*n*: patient number of subgroups; Complication: patient number with ophthalmic complication; aOR: adjusted odds ratio; C.I.: confidence interval. * The subgroup of the patients never using HCQ. ^†^ Adjusted for HCQ, hypertension, hyperlipidemia, rheumatoid arthritis, ankylosing spondylitis, systemic lupus erythematosus, and Sjogren’s syndrome.

## Data Availability

The data presented in this study are available on request from the corresponding author.

## Supplementary Materials

The following are available online at https://www.mdpi.com/article/10.3390/ijerph18158154/s1. Figure S1: Flowchart of type 2 diabetes patient enrollment with and without ophthalmic complications in the sensitivity analysis, Table S1: Demographic characteristics of diabetic patients in the sensitivity analysis, Table S2: Conditional logistic regression of risk of ophthalmic complications in the sensitivity analysis.
