# Supplementary material for: Relationship of Hydroxychloroquine and Ophthalmic Complications in Patients with Type 2 Diabetes in Taiwan
Source: Int J Environ Res Public Health. 2021 Aug 1;18(15):8154. doi: 10.3390/ijerph18158154 (PMC8345959; doi:10.3390/ijerph18158154)
Supplement: Supplementary file 1 [file ijerph-18-08154-s001.zip › ijerph-1264990-supplementary.pdf]

Supplementary Materials:

The sensitivity analysis with the matching score including hypertension and hyperlipidemia

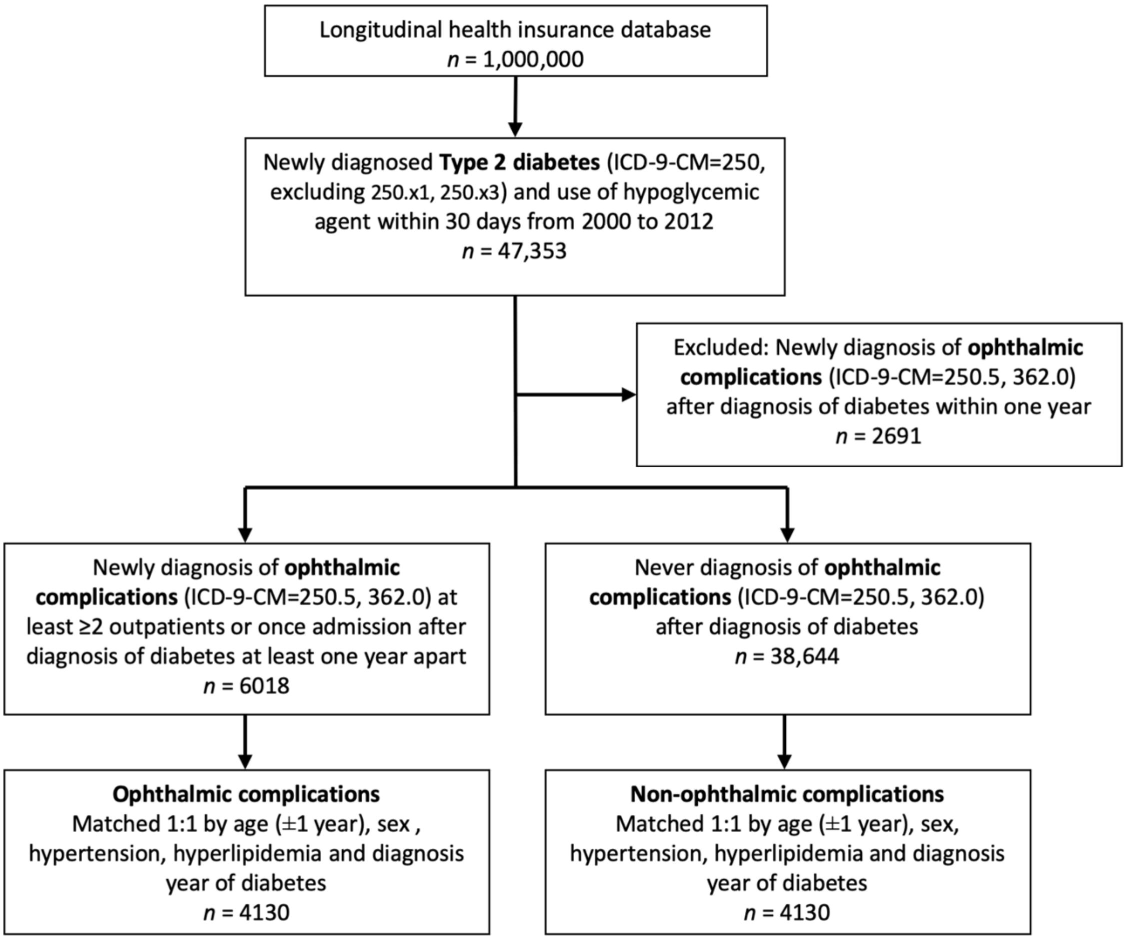

Figure S1. Flowchart of type 2 diabetes patient enrollment with and without ophthalmic complications in the sensitivity analysis.

Table S1. Demographic characteristics of diabetic patients in the sensitivity analysis.

	Case		Control		
Variables	*n*	%	*n*	%	*p*-value
Total	4130		4130		
Subgroups					
HCQ	78	1.9	68	1.6	0.404
Age					0.449
<40	95	2.3	94	2.3	
40-65	2697	65.3	2644	64.0	
≥65	1338	32.4	1392	33.7	
Mean ± SD	60.2 ± 10.6		60.4 ± 10.5		0.563
Sex					1
Female	2032	49.2	2032	49.2	
Male	2098	50.8	2098	50.8	
Hypertension	2756	66.7	2756	66.7	1
Hyperlipidemia	2183	52.9	2183	52.9	1
Rheumatoid arthritis	59	1.4	70	1.7	0.329
Ankylosing spondylitis	15	0.4	15	0.4	1
Systemic lupus erythematosus	3	0.1	6	0.1	0.317
Sjogren's syndrome	37	0.9	27	0.7	0.210

n: sample size; HCQ: hydroxychloroquine; SD: standard deviation.

Table S2. Conditional logistic regression of risk of ophthalmic complications in the sensitivity analysis.

Variables	cOR	95% C.I.	p-value	aOR†	95% C.I.	*p*-value
HCQ						
No	Reference			Reference		
Yes	1.15	0.83-1.61	0.398	1.21	0.86-1.72	0.278
Rheumatoid arthritis	0.83	0.58-1.19	0.318	0.78	0.54-1.13	0.197
Ankylosing Spondylitis	1.00	0.49-2.05	1.000	1.01	0.49-2.07	0.975
Systemic Lupus Erythematosus	0.50	0.13-2.00	0.327	0.43	0.10-1.75	0.236
Sjogren's syndrome	1.37	0.83-2.25	0.213	1.39	0.84-2.30	0.204

cOR: cude odds ratio; aOR: adjusted odds ratio.

†Adjusted for HCQ, rheumatoid arthritis, ankylosing spondylitis, systemic lupus erythematosus, and Sjogren's syndrome.
